# Antiviral Activity of Exopolysaccharides Produced by Lactic Acid Bacteria of the Genera *Pediococcus*, *Leuconostoc* and *Lactobacillus* against Human Adenovirus Type 5

**DOI:** 10.3390/medicina55090519

**Published:** 2019-08-22

**Authors:** Liubov Biliavska, Yulia Pankivska, Olga Povnitsa, Svitlana Zagorodnya

**Affiliations:** Zabolotny Institute of Microbiology and Virology, National Academy of Sciences of Ukraine, 03143 Kyiv, Ukraine

**Keywords:** exopolysaccharides, lactic acid bacteria, human adenovirus type 5, antiviral activity, cell cycle

## Abstract

*Background and objectives*: The use of antagonistic probiotic microorganisms and their byproducts represents a promising approach for the treatment of viral diseases. In the current work, the effect of exopolysaccharides (EPSs) produced by lactic acid bacteria from different genera on the structural and functional characteristics of cells and the development of adenoviral infection in vitro was studied. *Materials and Methods:* Cytotoxicity of six EPSs of lactic acid bacteria of the genera *Lactobacillus*, *Leuconostoc* and *Pediococcus* was determined by MTT (3-(4,5-dimethylthiazol-2-yl)-2,5-diphenyl tetrazolium bromide) assay. The influence of the EPSs on the infectivity of human adenovirus type 5 (HAdV-5) and on the cell cycle under a condition of adenovirus infection was studied using plaque reduction assay and flow cytometric analysis, respectively. *Results*: It was shown that exopolysaccharides were non-toxic to Madin-Darby bovine kidney cells (MDBK) as they reduced their viability by 3–17%. A change in the distribution of the cell cycle phases in the non-infected cell population treated with EPSs was observed. The analysis demonstrated an increase in the number of cells in the S phase by 47% when using EPSs 15a and a decrease in the number of cells in the G1 phase by 20–27% when treated with the EPSs 15a, 33a, and 19s. The use of EPSs did not led to the normalization of the life cycle of HAdV-5 infected cells to the level of non-infected cells. The EPSs showed low virucidal activity and reduced the HAdV-5 infectivity to 85%. Among the studied exopolysaccharides, anti-adenovirus activity was found for EPS 26a that is produced by *Lactobacillus* spp. strain. The treatment of cells with the EPS following virus adsorption completely (100%) suppressed the formation and release of HAdV-5 infectious. *Conclusions:* EPS 26a possessed distinct anti-HAdV-5 activity and the obtained data demonstrate the potential of using exopolysaccharides as anti-adenoviral agents.

## 1. Introduction

Despite successes of vaccination, chemoprevention, and chemotherapy of human respiratory viral infections, these diseases still are one of the leading causes of human infectious disorders. Adenoviruses cause keratoconjunctivitis, as well as respiratory, urogenital and intestinal tract infectious diseases. In addition, adenoviruses may persist in the lymphoid tissue of the tonsils and adenoids for a long time, causing upper respiratory tract infections in adults [1,2].

Various compounds have been found to be effective against adenoviruses. Anti-adenoviral activity was shown in vitro for nucleotide analogs [3,4]. Ribavirin, which affects many DNA and RNA-containing viruses, was found to be effective, partially effective, or not effective against adenoviral infection. Ganciclovir, which is efficient against cytomegalovirus infection, is effective against adenoviruses both in vitro and in vivo [5]. Cidofovir, which is characterized by a broad spectrum of antiviral activity, is used for the treatment of severe adenoviral infections [6]. Ribavirin, ganciclovir, and cidofovir are more or less successfully used for the treatment of severe human adenoviral pathologies, including hepatitis, cystitis, and pneumonia in immunocompromised organ transplant recipients [7,8]. At the same time, apparent adverse effects and the ability of adenoviruses to form resistant strains limit the use of these drugs [9]. Some chemically distinct compounds, including lipids, acridones, and imidazoquinolinamines, as well as other analogs of nucleosides, also show anti-adenoviral activity. Virus reproduction in cell cultures may be inhibited by high concentrations of green tea catechins [10]. Interferon drugs used to treat respiratory infections are most useful for prevention and cannot be considered as the primary medications for the treatment of severe infection. As adenoviruses have effective mechanisms that suppress the interferon-induced antiviral cascade, they are resistant to the action of interferon and its inducers [11].

Therefore, the development of new antiviral drugs that would be effective against adenoviruses without adverse effects, for a wide range of patients, including long-term users, is very urgent.

Currently, the use of probiotic antagonist microorganisms and their metabolic products represents a promising approach for the treatment of viral diseases [12]. Lactic acid bacteria (LAB), which can be found in the microbiota of the mucous membranes of the nose and mouth, and the gastrointestinal and urinary tracts, have a positive effect on these ecosystems as they stimulate the immune system and increase resistance to infections of bacterial and viral origin. LAB are the sources of multiple biologically active substances that vary in chemical structure and action spectrum [13]. During metabolic processes, lactic acid bacteria produce vital substances, including vitamins, amino acids, enzymes, fatty acids, exopolysaccharides, lactic acid, bacteriocins, and hydrogen peroxide, and significantly improve the intestinal absorption of micro- and macro-elements essential for the organism. It was also shown that exopolysaccharides that are secreted during the cultivation of LAB, in addition to their probiotic activity, can act selectively on pathogenic microbes and viruses without significant disturbances of the microbiocenosis and the development of inflammatory processes that occur during the use of broad-spectrum antibiotics and chemical antiviral agents [14]. As a result, exopolysaccharides can be used as a base for the production of harmless, non-toxic and acellular probiotic products and drugs that have a targeted therapeutic effect.

The present study aimed to investigate the antiviral activity of exopolysaccharides produced by lactic acid bacteria of the genera *Pediococcus*, *Leuconostoc* and *Lactobacillus* against human adenovirus type 5.

## 2. Materials and Methods

### 2.1. Virus and Cell Culture

In this study MDBK cells were obtained from the tissue culture collection of the Institute of Virology of the Bulgarian Academy of Sciences (Sofia, Bulgaria) and HAdV-5 was obtained from the collection of the Institute of Microbiology of the Budapest University of Medical Sciences (Budapest, Hungary) and propagated in HEp-2 (ATCC CCL-23) cells.

MDBK cells were maintained in sterile plastic falcon ^®^ (Sarstedt AG & Co. KG, Nümbrecht, Germany) in a growth medium composed of 45% DMEM (Sigma-Aldrich, St. Louis, MO, USA), 45% RPMI 1640 (Sigma-Aldrich, St. Louis, MO, USA) and 10% fetal bovine serum (FBS, Sigma-Aldrich, St. Louis, MO, USA) heat inactivated at 56 °C with antibiotics penicillin (Sigma-Aldrich, St. Louis, MO, USA) and streptomycin (100 μg/mL) (Sigma-Aldrich, St. Louis, MO, USA).

Cultivation, purification, and preservation of HAdV-5 were performed according to the standard method [15]. The infectious titer of the virus in MDBK cell culture was 7.0 × 10^7^ PFU/mL.

### 2.2. Tested Substances

Exopolysaccharides (EPSs) produced by lactic acid bacteria of the genera *Pediococcus*, *Leuconostoc* and *Lactobacillus* were extracted, purified and accumulated by the researchers from the Department of Physiology of Industrial Microorganisms at the Zabolotny Institute of Microbiology and Virology National Academy of Sciences of Ukraine (Table 1) [16,17]. Ribavirin (Rib) (Sigma-Aldrich, St. Louis, MO, USA) was used as a reference compound.

### 2.3. Cytotoxicity Assays

MTT-assay was used for the analysis of cell viability [18]. After 24 h of growth in growth medium, monolayers of MDBK cells in 96-multiwell plates were incubated with EPS at the concentrations of 375, 750 and 1500 μg/mL. Control cells were incubated with fresh medium lacking EPS for 72 h. A total of 20 μL of MTT solution 3-(4,5-dimethylthiazol-2-yl)-2,5-diphenyl tetrazolium bromide (Sigma-Aldrich, St. Louis, MO, USA) was added into wells and cells were incubated at 37 °C and 5% CO_2_ for 3–4 h, then the medium was removed and 150 µL of 96% ethanol was added. The plates were read using a Multiskan FC (Thermo Scientific, Waltham, MA, USA) with a 538-nm test wavelength.

Percentage decrease of cell viability under the condition of EPS action was calculated by the following formula:

% decrease of cell viability = 100 − (A/B × 100)(1)

where A is the mean optical density of the studied samples at a certain concentration, and B is the mean optical density of the control cell samples.

EPS concentration at which cell viability was inhibited by 50% (CC_50_) was estimated in comparison to the control cells not treated with EPS.

### 2.4. Cell Cycle Analysis by Flow Cytometry

MDBK cells infected with adenovirus (treated and not treated with the EPSs) were fixed in 96% ice-cold ethanol for 1 h, resuspended in 500 mL solution of phosphate-buffered saline (Sigma-Aldrich, St. Louis, MO, USA) that contained RNAse (100 μg/mL) (AppliChem GmbH, Darmstadt, Germany) and propidium iodide (PI) (50 μg/mL) (Sigma-Aldrich, St. Louis, MO, USA), and incubated at room temperate in a dark place for 1 h [19]. The cell fluorescence intensity was measured by a flow cytometer (Beckman Coulter Epics LX, Minneapolis, MN, USA) with a laser wavelength of 488 nm. A total of 20,000 cells were sorted per sample. Cell cycle profiles were analyzed with the program Flowing Software, v. 2.5 (Cell Imaging Core, Finland).

### 2.5. Antiviral Assay

To investigate the best order of EPSs’ antiviral effects, three experimental procedures were used as follows:

#### 2.5.1. Pre-Treatment of Cells with EPSs

To MDBK cells were added 100 μL of EPSs at the concentrations of 20, 100 and 500 μg/mL, which were then incubated at 37 °C for 24 h. Then, cells were infected with HAdV-5 (the multiplicity of the infection (MOI) of 7 PFU per cell). After 1.5 h, the virus-containing medium was removed, and 200 μL of serum-free medium was added.

#### 2.5.2. Co-Incubation of EPSs and HAdV-5

Adenovirus (MOI 70 PFU/cell) was mixed with an equal volume of EPS at a concentration of 1500 µg/mL and incubated at 37 °C for 3 h. The MDBK cell monolayer was infected with 50 µL per well of suspension of EPS-HAdV-5 of the appropriate dilution. Following the virus adsorption that was carried out at 37 °C for 1.5 h, the virus-containing material was removed, and 200 μL of serum-free medium was added.

#### 2.5.3. EPSs Were Added Post-Infection

The MDBK cells were first infected with HAdV-5 at 50 μL/well (MOI 3.5 PFU/cell), and the virus was adsorbed at 37 °C for 1.5 h. Then, the virus was removed, and the growth medium with the appropriate EPSs concentration was added (20, 100 and 500 μg/mL).

Plates with cells were kept at 5% CO_2_ at 37 °C until a visible cytopathic effect of adenovirus appeared (3–5 days), and the virus material was selected for further investigation of the virus titer. Non-treated cells and cells treated with HAdV-5 served as controls.

### 2.6. Virus Titration

The titer of the virus was determined by the plaque method, based on the formation of necrotic sites in the infected cells due to the reproduction of the virus. Cells were grown in a 24-well plate (TPP, Trasadingen, Switzerland) to form a 100% monolayer and were infected with serial ten-fold diluted lysates of cells previously infected with adenovirus (untreated or treated with different concentrations of EPSs) at 0.3 mL per well and incubated for 1.5 h at 37 °C at 5% CO_2_. After adsorption of the virus, the medium was removed, and the cells were covered with 1% methylcellulose (Sigma-Aldrich, St. Louis, MO, USA), DMEM medium (Sigma-Aldrich, St. Louis, MO, USA) and 2% fetal bovine serum (Sigma-Aldrich, St. Louis, MO, USA). Cells were incubated at 37 °C at 5% CO_2_ for 5 days. Next, the cover was removed and 200 μL of 0.2% crystal violet (Sigma-Aldrich, St. Louis, MO, USA) in 20% ethanol was added to the cell monolayer [20,21]. The titer of the virus was determined by the highest dilution of the virus in which the virus-induced plaques were formed according to the formula:

Virus titer PFU/mL = A/(B × C)(2)

where A is the number of plaques, B is the dilution of the virus, and C is the volume of the inoculum.

The antiviral activity of EPSs was determined using the following formula:

% inhibition = (1 − Virus titer (experiment)/virus titer (control)) × 100%(3)

A decrease in the infectious titer of the virus by 2 lg or more (or 99% or more), compared with the control, indicates significant activity of the compound against the virus, 97–99% demonstrates a moderate effect and less than 97% shows a relative activity [22].

### 2.7. Statistical Analysis

Statistical data processing was performed according to standard approaches for calculating statistical errors (standard deviation) using Microsoft Excel 2010. The results were expressed as mean *±* S.D. for three independent experiments. The Student’s *t*-test (unpaired, two-sided *t*-test) was used to evaluate the difference between the test sample and control. A *p* value of <0.05 was considered statistically significant.

## 3. Results

### 3.1. The Effect of Lactic Acid Bacteria Exopolysaccarides on Viability and Mitotic Activity of MDBK Cells

The issue of directed inhibition of the reproduction of infectious disease pathogens requires a search for medicines that are characterized by low toxicity and a broad spectrum of antiviral activity. As viruses are intracellular parasites, selective suppression of virus reproduction without adverse effect on the viability of host cells is one of the requirements for antiviral inhibitors.

**Figure 1:**
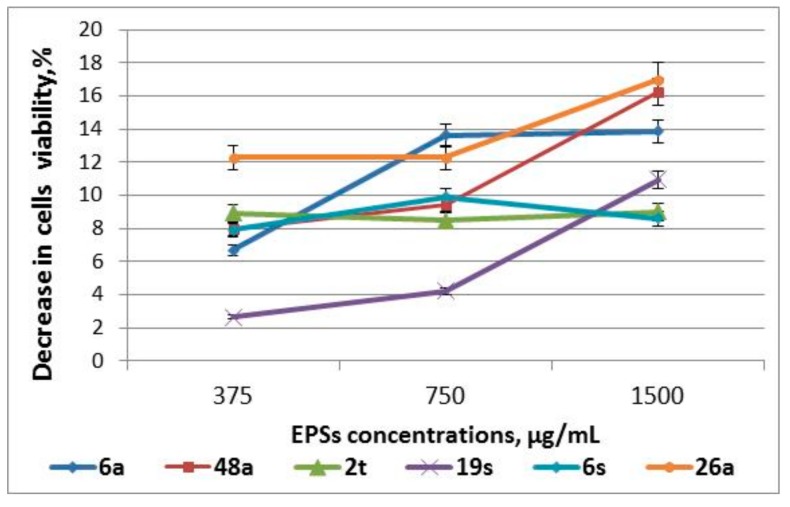
Cytotoxicity of exopolysaccharides in MDBK cells. Serial three-fold dilutions of EPSs in DMEM-RPMI medium were added onto the monolayer of MDBK in a 96-well plate for 72 h at 37 °C. Cell viability effect was determined by MTT assay. Values represent the mean ± S.D. for three independent experiments.

MTT analysis is one of the most simple and available methods for the estimation of cytotoxicity. The technique enables the identification of the negative impact of EPSs on the viability of cells and the functional activity of mitochondria (Figure 1).

It was found that EPSs at a concentration of 375–1500 µg/mL were not toxic for MDBK cells, as they suppressed their viability only by 3–17%, whereas the CC_50_ value was significantly higher than 1500 µg/mL. The CC_50_ value for referent compound Ribavirin was 400 µg/mL.

As toxic compounds often cause the termination of cell growth and proliferation, the effect of EPSs on the MDBK cell populations was studied. Cells were fixed and dyed with fluorochrome propidium iodide and analyzed using flow cytometry. The approach allowed the estimation of the distribution of cells by their structure and cell cycle phases, as well as the identification of apoptotic cells, as the propidium iodide signal is directly proportional to DNA content.

**Figure 2:**
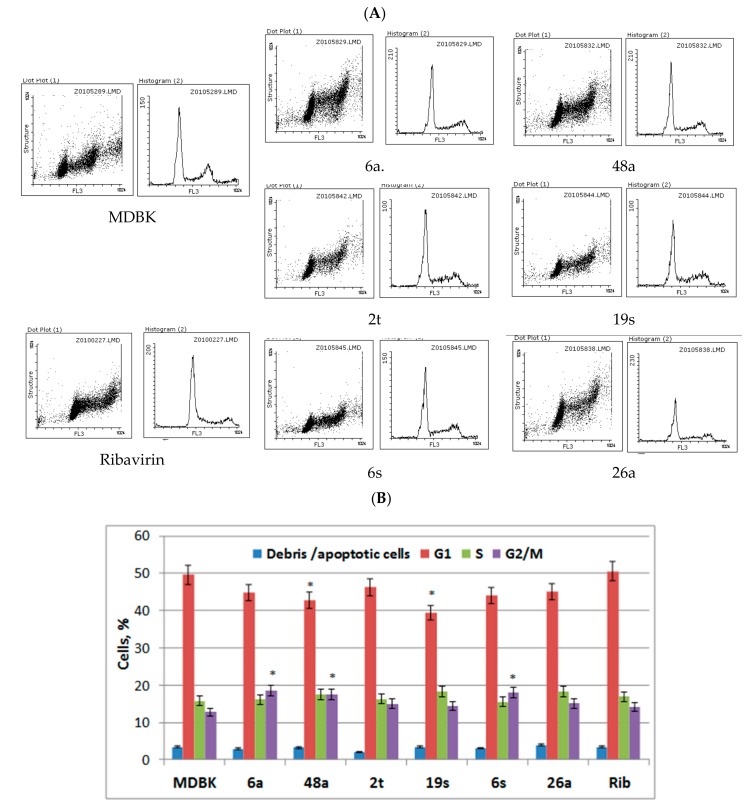
Influence of the EPSs on the cell cycle of the MDBK cells. (**A**) Cell cycle profiles of the control cells and EPS-treated cells (concentration of exopolysaccharides and ribavirin are 1500 and 125 µg/mL, respectively). (**B**) Cell cycle features after treatment with the EPSs were measured by flow cytometry following cell fixation and propidium iodide staining. Cell cycle profiles were analyzed with the Flowing Software, version 2.5. Results corresponding to the percentage of cells in G1, S, and G2/M phases of three independent experiments are presented as mean ± S.D. * Significant difference between test sample and control cells (*p* < 0.05).

As can be seen from cell cycle profiles (Figure 2A) based on the distribution of cells according to the structure and cell cycle phase, the histograms of EPS-treated and control MDBK cells are similar but not identical. It was revealed that after 48 h of growth, 50% of control MDBK cells remained in the G1 phase, 16% were in the S phase, and 13% were in the G2/M phases (Figure 2B).

Under the conditions of the compound treatment, the distribution of cells in G1 and G2/M phases was similar to control cells. However, compared to control cells, EPSs 48a and 19s decreased the number of cells in the G1 phase up to 16%, whereas EPSs 6a, 48a, and 6s increased the number of cells in the G2/M phase by 11–42%.

### 3.2. Anti-Adenoviral Activity of EPSs

The anti-HAdV-5 activity of EPSs (in non-toxic concentrations) was confirmed by measuring virus yield synthesized de novo using a plaque reduction assay.

**Figure 3:**
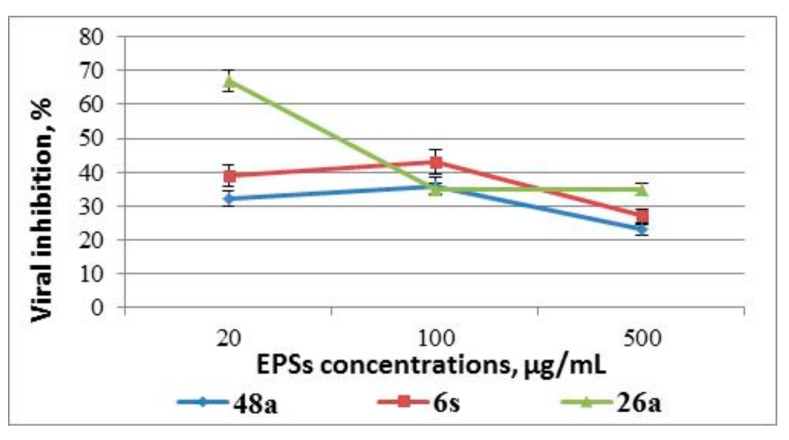
Effect of EPSs on infectivity of adenoviral offspring: the results of the analysis of the EPS-mediated antiviral effect for pre-treatment of cells with EPSs. Results are presented as percentage of infectious virus titer reduction and are the mean of three independent experiments.

Analysis of the antiviral activity of EPSs added to MDBK cells 24 h before adenovirus infection demonstrated that only EPSs 48a, 26a and 6s showed low antiviral activity, reducing virus reproduction by 23–67% (Figure 3), indicating the inefficiency of EPS use according to the suggested treatment approach.

**Figure 4:**
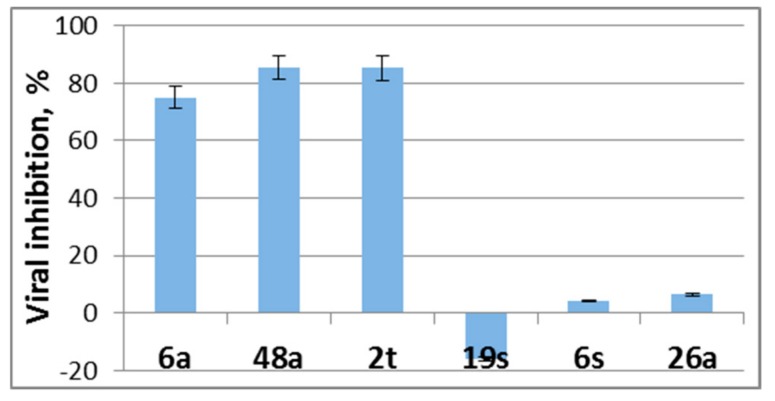
Influence of EPSs on the formation of infectious progeny of adenovirus. The results of the analysis of the EPS-mediated antiviral effect for co-incubation of EPSs (concentration 1500 µg/mL) and HAdV-5. Results are presented as percentage of infectious virus titer reduction and are the mean of three independent experiments.

It was found that EPS showed weak virucidal activity decreasing the infectious titer of HAdV-5 by 3–85%, whereas incubation of the virus with EPS 6a increased HAdV-5 reproduction by 15% (Figure 4).

**Figure 5:**
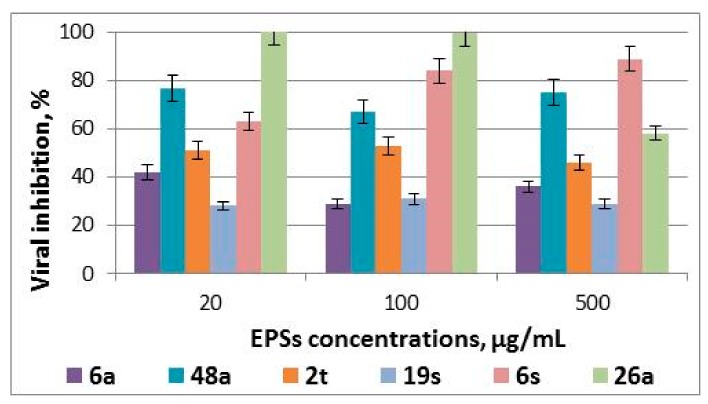
Effect of EPSs on adenovirus reproduction: the results of the analysis of the antiviral activity of EPSs added after infection. Results are presented as percentage of infectious virus titer reduction and are the mean of three independent experiments.

Using EPSs at late stages of adenovirus reproduction and immediately after virus adsorption, it was found that only EPS 26a shows apparent anti-HAdV-5 activity. At the concentrations of 20 and 100 µg/mL, the EPS absolutely blocked the synthesis of viral progeny (Figure 5). Other EPSs used in the analyzed concentrations reduced the infectious titer of the virus by 18–93%. The use of Ribavirin at concentrations of 32–125 μg/mL after infection of the cells with adenovirus resulted in a decrease in HAdV-5 reproduction by 96.3–99.8% (data not shown).

Virus infection frequently results in the disturbance of key cellular processes within the host cell. The subversion of cell cycle pathways is a well-established mechanism by which viruses create the most suitable environment for their replication [23]. Notably, the induction of the S-phase is either mandatory or at least advantageous for lytic replication of a number of viruses. Adenoviral infection has been reported to have effects on the cell cycle. It is well-known that adenoviral E1 gene products interact with pRb (retinoblastoma protein), causing the release of E2F transcription factor, which potentiates transition from the G1 to the S phase, in which productivity is greatest. HAdV infection of a range of epithelial cell lines, including a primary cell line, causes G2 phase synchronization and cell cycle arrest [24,25]. This synchronization in the G2 phase may be a significant factor contributing to the cell-size increase [25]. Therefore, the influence of EPSs on the cell cycle under the conditions of adenovirus infection was analyzed using flow cytometric analysis.

**Figure 6:**
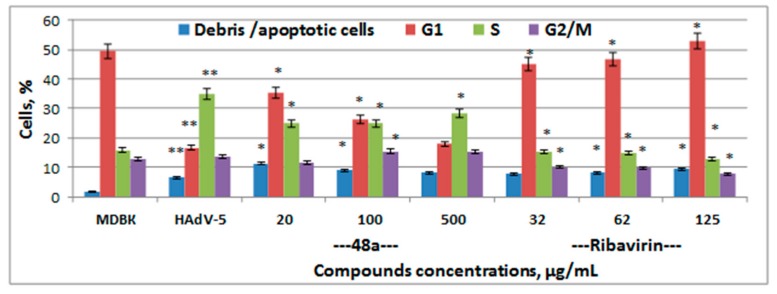
Influence of the EPSs on the cell cycle of the MDBK in the presence of adenovirus infection. The cells’ fluorescence intensity was measured by a flow cytometer (Beckman Coulter Epics LX, MN, USA) with laser wavelength 488 nm. Cell cycle profiles were analyzed with the program Flowing Software, version 2.5. Results corresponding to the percentage of cells in the G1, S, and G2/M phases of three independent experiments are presented as mean ± S.D. * Significant difference between test sample and control of infected cells (*p* < 0.05). ** Significant difference between a control of infected cells and control cells *(p* < 0.05).

Significant changes in the cell cycle of cells infected with HAdV-5 compared to non-infected cells were revealed. In particular, the number of cells in the G1 phase decreased by 67%, whereas the number of cells in the S phase is doubled (Figure 6). The reduction of the cell population in G1 phases demonstrated the suppression of the transition of cells through the mitotic phase. As infected cells enter the S phase and the G2/M phase is blocked, cells produce viral DNA, late viral proteins and virions. Further, these cells are destroyed and detach from the monolayer.

It was found that after the treatment of cells infected with adenovirus using exopolysaccharides, the distribution of cells in cell cycle phases was similar to the distribution of cells infected with adenovirus, demonstrating the inefficiency of the exopolysaccharide treatment (data not shown). A decrease in the number of cells in the S phase by 5–33% compared with the distribution of infected cells was observed. However, there was no increase in the number of cells in the G1 phase, indicating continued blocking of the mitotic phase of the cell cycle due to viral infection. Only the use of EPS 48a at a concentration of 100 and 20 µg/mL resulted in a significant increase in the number of cells in the G1 phase (1.5–2 times) compared with infected cells, and a decrease in the number of cells in the synthetic phase by 18–29%, indicating normalization of the cell cycle of infected cells to the level of uninfected cells (Figure 6).

## 4. Discussion

In recent years, there has been a tendency to use LAB exopolysaccharides for the treatment of infections of the respiratory tract, gastrointestinal tract, and urinary system, as well as cancer, allergic diseases, and viral infections [26]. However, despite the wide range of applications of lactic acid bacteria exopolysaccharides, their role in living organisms and their biological activities are not fully understood. The production of exopolysaccharides (EPS) by lactic acid bacteria (LAB) has been intensively studied, and a significant amount of data describing their composition, structure, and properties have been accumulated. EPS producing strains were found among representatives of the genera *Streptococcus, Lactococcus, Lactobacillus, Leuconostoc, Pediococcus,* and *Weissella*. LAB exopolysaccharides have unique physical and rheological properties. As a result, they are used in the food industry as binding, stabilizing, and gel-forming agents, specifically in the production of dairy products. In recent years, much attention has been paid to the study of the biological activity of EPSs. In particular, it was shown that they have immunostimulating, antitumor, and antioxidant qualities. Since the biological activity of EPSs is strain-specific, the search for new strain-producers among LAB representatives found in the natural microbiota of fermented products is of great interest. Strains producing EPSs, especially in high amounts, are important for improving the rheological properties of the product and may possess possible health-improving effects for the human body. Previously, searches for LAB strains producing exopolysaccharides were performed. The isolates of LAB present in traditional fermented milk products, fermented fruits and vegetables were analyzed. Most EPS producers were found among representatives of the genera *Lactobacillus, Leuconostoc* and *Pediococcus* isolated from pickled apples, pickled tomato juice, and sauerkraut. Therefore, the biological activity of these EPSs was analyzed in the current work. In the preclinical study of potential drugs, the first step is associated with the assessment of the compound’s toxicity for cell culture (namely, the study of the effect of different concentrations on the structural and functional properties of the cells). It should be noted that most toxic agents act on the cell by interfering with the molecular mechanisms of homeostasis. This action can be expressed in a whole set of effects, which include changes in the fundamental cellular reactions, leading to destructive effects. We have studied the impact of EPSs extracted from lactic acid bacteria of the genera *Pediococcus*, *Leuconostoc* and *Lactobacillus* on the growth and division of MDBK cells sensitive to adenovirus. It was identified that EPSs in the studied concentrations are not toxic to MDBK cells, since they reduce cell viability only up to 17%. Also, the analysis of the effect of the highest EPS concentration on the cell cycle of MDBK cells showed the absence of significant changes in the distribution of the cell population in the G1, S and G2/M phases of the cycle. The obtained data allowed the use of all EPSs for the study of their antiviral potential. It is known that bacterial exopolysaccharides show significant antiviral activity due to the degradation of the viral particles, a decrease in the titer of viruses, the blocking of viral DNA replication, and the release of the infectious virus particle. However, the antiviral potential of these drugs has not been studied sufficiently. As a result, to investigate and exhibit EPS-mediated anti-adenoviral activity and to determine the stage of viral reproduction inhibited by EPS, various EPS treatment schemes were used in the work. Based on three different treatments, the results suggested that EPSs have a specific inhibitory effect on HAdV-5. The EPSs showed low virucidal activity and reduced HAdV-5 infectivity up to 85%. In most cases, neither pre-treatment resulted in a significant inactivation of virion infectivity. The inhibitory effects were observed only when EPSs were added to cells at the end of the virus adsorption period. However, only EPS 26a (produced by *Lactobacillus* sp.) reduced the titer of the virus obtained de novo and inhibited HAdV-5 plaque formation by 100%.

Currently, several possible mechanisms of the influence of lactic acid bacteria and their metabolites on the development of viral infection have been identified: i) inhibition of virus adsorption and penetration into cells as a result of direct bacterium/metabolite-virus interaction; ii) inhibition of late stages of viral reproduction and reduction of their infectivity; and, iii) stimulation of the immune system [26]. As a result, it was shown that probiotics can "capture" the virus of vesicular stomatitis (VSV) by direct interaction between LAB cells (*L. paracasei A14, L. paracasei F19, L. paracasei/rhamnosus Q8, L. plantarum M1.1* and *L. reuteri DSM12246*) and the lipid envelope of the VSV, which leads to blocking of the virus adsorption on the cell. Similar data were shown for *E. faecium NCIMB 10415* against influenza viruses and *L. gasseri CMUL57* isolated from vaginal microbiota against the herpes simplex virus type 2 (HSV-2). The probiotics of lactic acid bacteria *Lactobacillus* and their exopolysaccharides can stimulate the synthesis and accumulation of interleukin 12 to enhance the activity of natural killer cells and the synthesis of IgA in the spleen. Such activation of Th1 immune responses and the production of IgA determine their action against influenza [27]. In addition to direct interaction between viruses and LAB, bacteria synthesize some metabolites with antiviral activity. As a result, hydrogen peroxide, which is produced by *Lactobacillus* sp., plays an essential role as a natural microbocide within the vaginal ecosystem and is toxic to human immunodeficiency virus type 1 (HIV-1) and HSV-2. Lactic acid, which is the final product of carbohydrate metabolism and is synthesized by all types of lactobacilli, is essential for maintaining the required pH of the genital organs, since it is known that the acidic pH of the environment inactivates HIV, and HSV-1 and -2. It has been revealed that lactobacilli can produce compounds that inhibit replication of the viruses. The *L. brevis* cell wall inhibits the replication of HSV-2 DNA in cell culture, while the acidic products of the *Lactobacillus* metabolism inhibit the activation of T-lymphocytes, leading to a decrease in the sensitivity of lymphocytes to HIV infection, which is especially important for mixed HIV–HSV infections [28]. It has been established that some types of bacteriocins and exopolysaccharides have an apparent virucidal effect on influenza, HSV-1 and -2, HIV and Newcastle disease.

The characteristic changes in DNA synthesis and content induced by HAdV-5 infection allow the use of flow cytometry to detect not only viral infection but also potential antiviral activities [25]. The influence of the EPSs on the cell cycle under a condition of adenovirus infection was studied using flow cytometric analysis of propidium iodide-stained cells. The use of EPSs did not lead to the normalization of the life cycle of HAdV-5 infected cells to the level of non-infected cells, as the number of cells in the S phase was not decreased significantly (except EPS 48a) and there was no transition of cells into the G1 phase, which indicates the blocking of the mitosis in infected cells.

Taken together, our data suggested that exopolysaccharides produced by LAB strains exhibited anti-HAdV-5 activity in vitro. Among the six used strains, one LAB strain (*Lactobacillus* sp.), which produced EPS 26a suppressing the yield of virus particles, was selected. Furthermore, this strain is interesting as potential probiotic or starter, whereas the in-depth investigation of the functional and technological properties, EPSs monosaccharide composition and structure are currently studied. 

## 5. Conclusions

In summary, the EPS 26a produced by *Lactobacillus* sp. possessed significant anti-HAdV-5 activity, based on the obstruction of HAdV-5 reproduction, inducing the formation of non-infectious virus progeny. Thus, this LAB strain is a promising producer of EPS with antiviral activity against HAdV-5. However, further investigation is needed to explore the antiviral mechanism of such an EPS in detail.

## Figures and Tables

**Table 1:** Sources used to extract exopolysaccharides (EPSs) and genera of lactic acid bacteria (LAB) producing respective EPS.

EPS	Source of Extracted EPS	Genus of Lactic Acid Bacteria
6a	pickled apples	*Pediococcus* sp.
48a	pickled apples	*Leuconostoc* sp.
2t	pickled tomato juice	*Leuconostoc* sp.
19s	sauerkraut	*Leuconostoc* sp.
6s	sauerkraut	*Leuconostoc* sp.
26a	pickled apples	*Lactobacillus* sp.
